# Book Reviews

**Published:** 1877-04

**Authors:** 


THE SURGERY SURGICAL PATHOLOGY AND SURGICAL ANATOMY OF THE FEMALE PELVIC ORGANS, In a series of colored plates taken from nature, with commentaries, notes and cases. By *Henry Savage, M. D., London, F. R. CS.*, One of the Consulting Medical Officers of the Samaritan Hospital; third edition, revised and greatly enlarged; an additional plate, also thirty-six wood engravings, with special illustrations of the operations on vesico-vaginal fistula ovariotomy and perineal operations. Philadelphia: Lindsay and Blakiston. 1876.
(For sale by subscription by *W. T. Keener*, Chicago.)

This elegant work has reached the third edition, a sure evidence of its great value to the profession. In this edition the anatomical plates are executed in the same beautiful manner as those of the two preceding editions. The accuracy of these plates connected with the lucid and comprehensive description accompanying them, render the book of Dr. Savage a useful guide to the student, and an acceptable office companion to the special practitioner.

While we willingly accord to it the highest praise as a work on the anatomy of the female pelvic organs, we must say that the surgery and surgical pathology of these organs are rendered in a very superficial and unsatisfactory manner. We believe in fact, the book would have done more credit to the author if he had confined himself to the anatomical features of the work so admirably given by him. While all the purely anatomical illustrations are excellent and instructive, a part if not the whole of the surgical and pathological plates are weak and tame in their effect, and some of them puerile. As one of this kind, we instance figure 1, in plate 14. About the only thing this figure illustrates is the utter impossibility of showing the operation for verico-vaginal fistula to a class of students in their seats.

Notwithstanding these defects, the good qualities ef "Savage on the Female Pelvic Organs," will continue to make a demand for the book.

Transactions of the Medical and Chirurgical Faculty of Maryland, at its 78th Annual Session. Held at Baltimore, April, 1876.

After the usual record of business, the address of Dr. Roberts Barthalow is given. His topic, the degree of certainty in therapeutics, is one interesting to all thinking medical men.

Dr. B. believes it to be criminal for any practitioner of medicine to be an unbeliever in the positiveness of therapeutic effects. He cites in support of his argument the effect of numerous remedies in certain forms of disease, as the specific effect of quinia in malarial fevers, and of twenty grain doses of this medicine arresting an acute inflammation; the antidotal power of mercury in constitutional syphilis; chloral as a soporific; morphia as a curer of pain.

Dr. Johnston gives quite an interesting record of cases of transfusion, and an operation to relieve vesical ectopic deformity. The patient, a boy, was eight years old. "The umbilicus was wanting, as were the pubic bodies; a deeply red oval area of oozing mucous membrane in the supra-pubic region represented the bladder; the small pores at its lower part were the opening of the ureters; the penis was split as if horizontally, and the urethra reduced to an uncovered groove, continuous with the vesical membrane.

Below was a small scrotum provided with diminutive testicles; while above, on either side, an inguinal hernia swelled with every effort of the subject. The red bladder measured 3½ inches in width by 2½ inches in height, with ample space above to the xiphoid cartilage.

On the 22d day of January, 1876, the operation was performed. A great umbilical flap was brought down over the bladder, the skin surface lying under, and this was covered by two wing flaps, one from either groin, which met in the middle line, and were secured to the first oblong flap. The space left by the umbilical flap was almost effaced by drawing together the integuments of opposite sides, and which had been dissected up; while the spaces left by the wing flaps were obliterated by a gliding of the skin which lay beyond the groins, made free underneath and attached to the outer wing flaps, now fixed in their new situation by silver and iron wire. As tension strained the lateral flaps, a crescentic incision was made beyond each groin to reduce it.

In about five weeks, he was able to return home greatly benefited, the operation having proven a success, perfect with the exception of a small loss of substance at the lower edge of the umbilical flap. In its present condition, the child will remain until cicatrization shall have completed its contraction, whereupon a second operation will be attempted, having for its object the formation of a sort of roof for his urethral groove.

As things are, however, the urine escapes over and around the penis only, and he is able to wear advantageously a rail-road urinal."

Dr. Van Bibber gives some cases of abdominal disease, one of which is very interesting. The case was first supposed to be one of constipation, but the various cathartics proving in-efficient, Dr. V. was called in consultation, when the diagnosis of intestinal obstruction was made. Electricity was now tried. A strong Faradic current was passed over the abdominal muscles. The operation was prolonged, with the full strength of the battery, for fifteen minutes. This being tried the second day, and proving ineffectual, an effort was made to insert a long tube beyond the sigmoid flexure of the colon. Whilst making the exploration for this purpose, Dr. V. detected lying in the hollows of the sacrum, a tumor, which felt like a bundle of intestines in a scrotal hernia. The diagnosis then made, was that a portion of the small intestines had fallen into the pelvis, and was there retained, thus causing the obstruction. An effort to inflate the intestinal canal with oxygen gas, was a failure, and the patient died soon afterwards.

In an autopsy, 36 hours after death, it was found that a portion of the ileum had been drawn into the pelvis during a previous attack of peritonitis, and was bound there by strong bands of lymph.

Dr. Gibbons reports a case of empyema cured by thoracen-tesis.

A report on obstetrics by Dr. Erich speaks highly of the use of fifteen grain doses of quinia to accelerate the contractions of the uterus in lingering labors.

Of the obstetrical binder, he says in case of post partum hemorrhage, it will change an internal, and consequently, concealed hemorrhage into an external one.

Dr. Conrad gives a carefully prepared article on Insanity, in its financial relation to the state.

This little volume contains much that is interesting, and most of the papers are excellent. The print, paper, and proof-reading are all good.

W. H. M.

Yellow Fever and Malarial Diseases, embracing a history of the Epidemics of Yellow Fever in Texas; new views on its diagnosis, treatment, propagation, and control; descriptions of Dengue, Malarial Fevers, Jaundice, the Spleen and its Diseases, and Diarrhœa Hemorrhagica; with practical remarks on their successful treatment, etc. By *Greensville Dowell, M. D., etc.* Philadelphia. 1876.

This book is an octavo of upwards of 240 pages. Its lengthy title indicates the scope of the discussion it contains. The discussion is everywhere searching and thorough. On Yellow Fever, Dr. D. speaks with authority, having treated over two thousand cases of this disease. Many of his ideas as to the pathology of the affections discussed are original, but hereafter no thorough study of these disorders as they occur in the South, can omit the work of Dowell, which must be stamped as standard.

